# Environmental risks to humans, the first database of valence and arousal ratings for images of natural hazards

**DOI:** 10.1038/s41597-022-01370-x

**Published:** 2022-06-14

**Authors:** Giulia Prete, Bruno Laeng, Luca Tommasi

**Affiliations:** 1 Department of Psychological, Health and Territorial Sciences, “G. d’Annunzio” University of Chieti-Pescara, Chieti, Italy; 2 Department of Psychology, University of Oslo, Oslo, Norway

**Keywords:** Natural hazards, Human behaviour

## Abstract

Due to their relevance for the entire society, environmental hazards have largely been investigated in terms of their psychological effects. However, a complete image database comprising different categories of catastrophes has not been proposed yet. We selected 200 photographs of the most frequent natural disasters with the aim to collect the emotional reactions of observers. In particular, 20 stimuli were selected for each of the following 10 categories: earthquake, volcanic activity, lightning, hailstorm, drought, fire, landslide, epidemic, and neutral and positive images as control categories. A sample of 605 participants completed an online survey, in which they were asked to rate either the valence or the arousal of each stimulus, by using a Self-Assessment Manikin. The Environmental Risk to Humans database associates the emotional reactions to these visual stimuli, together with the demographics of the sample (e.g., gender, age, marital status, income, previous experience of natural disasters). The database constitutes a tool to explore human reactions to natural hazards, providing a controlled set of stimuli for different types of catastrophes.

## Abstract (summary)

**Table:** 

Measurement(s)	valence and arousal
Technology Type(s)	human ratings
Factor Type(s)	natural hazards
Sample Characteristic - Organism	Homo sapiens
Sample Characteristic - Environment	anthropogenic environment

## Background & Summary

Every place on planet Earth is potentially subjected to environmental hazards, which can be defined as natural events happening with a specific frequency, and which can be responsible for direct and indirect costs for human beings. Each area of the Earth is categorized according to its specific environmental risk, even if our planet as a whole shares an overall risk level. As reported by the Centro euro-Mediterraneo per i Cambiamenti Climatici (CMCC) “*the* 2*0*2*0 Global Risks Report’s list of top risks for the likelihood of occurrence placed extreme weather, a failure to act on climate change, and natural disasters in the top three spots*” (https://www.climateforesight.eu/global-policy/global-risks-report-2020/). Similarly, The Global Risks Report 2020 highlighted that “*for the first time in the survey’s 10-year outlook, the top five global risks in terms of likelihood are all environmental*” (https://sdg.iisd.org/news/environmental-hazards-feature-in-2020-global-risk-report/).

According to the Emergency Events Database (EM-DAT, CRED/UCLouvain, Brussels, Belgium – www.emdat.be; D. Guha-Sapir), an online database describing the occurrence and the effects of thousands of natural disasters since 1900 (supported by the World Health Organisation and the Belgian Government), there are two main disaster groups which threaten human life: natural disasters and technological disasters. Within the category of natural disasters there are six areas: Biological, Geophysical, Climatological, Hydrological, Meteorological and Extra-terrestrial disasters (https://www.emdat.be/guidelines). Each of these areas contains specific natural events (e.g., earthquake) responsible for human deaths.

The severity of the effects of such natural hazards on human life is well known, so much so that an increasing amount of research is carried out to explore these effects on human health, social habits, policy-making and psychological effects. In this scenario, it is surprising to notice that no specific visual database exists, in which the emotional reactions of the observers to depicted natural hazards are categorized, and which could be made available to the research community working on the psychological and communicative aspects of disasters. Starting from this observation, we aimed to fill the gap by creating a freely accessible database of images showing the most frequent types of natural disasters. It has to be specified that some other databases have been proposed in this context, but they are either not freely accessible or focussed on one or some specific natural hazards (e.g., volcanic activities^1^), beyond lacking of a large sample validation^2^.

In this work, we started from an official taxonomy of natural disasters and selected a number of realistic photographs available online. Firstly, we selected 10 categories, including one neutral and one positive scenario, then, we accurately selected 20 photographs for each category and presented them to a large sample of observers. Half of the sample was asked to judge the emotional valence elicited by each image (from very negative to very positive valence), the other half was asked to indicate the arousal level induced by each image (from very low to very high arousal). We believe this database can offer a solid control over the emotional content of images of natural hazards used as stimuli, given the growing need to investigate human perception, reaction, adaptation and beliefs on these increasingly frequent events.

We started from the idea of selecting two events for each of the four areas included in the EM-DAT taxonomy which features the most common natural disasters, namely Geophysical, Climatological, Hydrological, Meteorological. In particular, the selected disasters were Earthquake and Volcanic activity (Geophysical), Lightning and Hailstorm (Meteorological), Landslide and Wave action (Hydrological), Drought and Fire (Climatological). However, due to the outbreak of the SARS-COV-2 pandemic^3^, we could not give up to include Epidemic as a Biological risk, so that we replaced Wave action with Epidemic (also due to the fact that images depicting the effects of waves are difficult to identify, because they can be easily exchanged with images depicting the effects caused by other disasters, such as earthquakes and landslides). Furthermore, since the aim of the database is to allow researchers interested in this topic to have a free set of standardized images, we also included two control categories: an emotionally neutral category and a positive category (depicted by means of daily life images, such as houses and roads for the Neutral category, or sunset over the sea and flowery meadow for the Positive category). As in previous studies in which emotional ratings were collected, we used a Self-Assessment Manikin (SAM) evaluation^4^ to collect both valence and arousal ratings.

## Methods

### Participants

Between October 2020 and April 2021, a total sample of 953 participants received and accepted an invitation to take part in an online survey. Participants were invited via email and social media (demographic information cannot be balanced *a priori*). Inclusion criteria, which were checked at the beginning of the survey, were the voluntary subscription to take part in the online survey and to be at least 18 years old. Data were considered (and reported here) only for those participants who rated all of the stimuli, for a final sample of 605 participants (399 females, 202 males, 4 participants did not report their gender). All demographic details are available in figshare Table 1 (to note that, due to the impossibility of an *a priori* selection of the sample, demographic features can differ among subsamples).

**Table 1:** Mean, Standard Error and Cronbach’s alpha values for the Valence and Arousal ratings of the 10 categories of stimuli.

	Valence			Arousal			Valence and Arousal correlations
	Mean	Standard Error	Cronbach’s alpha	Mean	Standard Error	Cronbach alpha	Pearson’s r (p values)
**Fire**	2.82	0.11	0.81	5.44	0.17	0.85	0.03 (0.70)
**Drought**	4.63	0.21	0.74	3.56	0.13	0.82	−0.01 (0.98)
**Volcanic activity**	4.48	0.10	0.91	5.48	0.11	0.90	0.03 (0.69)
**Earthquake**	2.07	0.05	0.89	6.31	0.09	0.90	0.11 (0.18)
**Hailstorm**	3.79	0.10	0.85	4.27	0.11	0.89	0.02 (0.83)
**Lightning**	4.71	0.07	0.93	5.27	0.12	0.89	−0.02 (0.83)
**Landslide**	2.97	0.17	0.78	5.41	0.16	0.83	0.05 (0.53)
**Epidemic**	3.17	0.10	0.85	5.03	0.12	0.87	−0.08 (0.35)
**Neutral**	5.33	0.19	0.75	2.73	0.08	0.81	0.07 (0.35)
**Positive**	7.91	0.06	0.87	2.82	0.06	0.91	0.21 (0.01)

The last column shows the correlation index (and the respective *p*-value) between valence and arousal ratings for each category of stimuli.

The total set of 200 stimuli was divided into two blocks, defined as i) Even - containing images with an even code, and ii) Odd - containing images with an odd code. Each participant was presented with one of the two blocks in order to avoid possible fatigue effects due to the length of the task, following the procedure used in other published databases (e.g.^4^,). Thus, four different links were created: two links were associated with the valence ratings (Valence-Even, Valence-Odd), the other two links were associated with the arousal ratings (Arousal-Even, Arousal-Odd). The Valence-Even subgroup was composed of 153 participants, including 110 females and 43 males; the Valence-Odd subgroup was composed of 117 participants, including 71 females and 46 males; the Arousal-Even subgroup was composed of 222 participants, including 154 females and 65 males (3 participants preferred not to indicate their gender); the Arousal-Odd subgroup was composed of 113 participants, including 64 females and 48 males (1 participant did not report gender). Each participant was also required to indicate: device used to complete the task (computer, tablet, smartphone, other), age range, educational qualification, current occupation, personal monthly income, family monthly income, marital status, number of children and country of residence. Ten participants lived in Germany, one in Belgium, one in Switzerland and all the remaining sample (N = 595) lived in Italy (for all the other demographic information see figshare Table 1). At the end of the task, participants were also asked to report if they had directly personally experienced one or more natural disasters in their life (hail, earthquake, avalanche, tsunami, landslide, flood, thunderstorm, fire, windstorm, volcanic eruption, drought, pandemic, or other). For each of these categories, they were required to report which effects the specific event had on their life (no direct effect; slight psychological, physical or material damage; significant psychological, physical or material damage; loss of one or more significant others). All these demographic and/or personal information are reported in figshare Table 1. The study was carried out in accordance with the Declaration of Helsinki and it was approved by the Institutional Review Board of Psychology (IRBP) of the Department of Psychological, Health and Territorial Sciences, University of Chieti (Protocol number: 20012).

### Stimuli and procedure

All data and stimuli are freely available on Figshare platform^5^. Stimuli were colour photographs downloaded from Flickr (https://www.flickr.com/photos/), which were selected only if they either had no copyright (CC0 1.0, public domain), or whether they were allowed to be shared and adapted by giving the appropriate credits (CC BY 4.0) and/or be redistributed under the same conditions as the owner (CC BY-SA 4.0). For each category of natural disaster, the first 20 images meeting these criteria and with acceptable resolution and size were selected. All of the photographs were manipulated in order to be resized (final size: 960 × 720 pixels), and some of them were also cropped, in order to obtain the same height-to-width ratio for all the stimuli. figshare Table 2 contains the complete list of all the photographs used, in .csv format, including the link to the original photo in Flickr, the name of the photographer, the original size and the specific kind of licence for each image. Search started by using as a keyword a specific natural event (e.g., earthquake) and 20 photographs were selected for each of the 10 categories. Original images were downloaded and resized, then they were saved in.jpg format. Contrast and lightness were not modified in order to leave each image as much in its original form as possible, presumably preserving the eye-witness documenting intentions of the authors.

**Table 2:** Valence scores correlations among the 10 categories of stimuli (*r* and *p* values): all comparisons are significant excepting for: Drought vs Earthquake, Hailstorm vs Epidemic, Neutral vs Volcanic activity, Earthquake and Epidemic, Positive vs Volcanic activity, Hailstorm and Lightning.

*Valence*	Fire	Drought	Volcanic activity	Earthquake	Hailstorm	Lightning	Landslide	Epidemic	Neutral	Positive
**Fire**		0.3238	0.4371	0.7036	0.4242	0.3693	0.6744	0.4347	0.1804	−0.2394
		p < 0.001	p < 0.001	p < 0.001	p < 0.001	p < 0.001	p < 0.001	p < 0.001	p = 0.026	p = 0.003
**Drought**	0.3238		0.4448	0.1177	0.4176	0.4589	0.3067	0.2715	0.5070	0.3069
	p < 0.001		p < 0.001	p = 0.148	p < 0.001	p < 0.001	p < 0.001	p = 0.001	p < 0.001	p < 0.001
**Volcanic activity**	0.4371	0.4448		0.2767	0.2912	0.7457	0.2872	0.2684	0.0669	0.0140
	p < 0.001	p < 0.001		p = 0.001	p < 0.001	p < 0.001	p < 0.001	p = 0.001	p = 0.412	p = 0.864
**Earthquake**	0.7036	0.1177	0.2767		0.2360	0.2513	0.8192	0.4043	−0.0986	−0.5306
	p < 0.001	p = 0.148	p = 0.001		p = 0.003	p = 0.002	p < 0.001	p < 0.001	p = 0.226	p < 0.001
**Hailstorm**	0.4242	0.4176	0.2912	0.2360		0.4107	0.3864	0.1531	0.3969	0.0583
	p < 0.001	p < 0.001	p < 0.001	p = 0.003		p < 0.001	p < 0.001	p = 0.059	p < 0.001	p = 0.474
**Lightning**	0.3693	0.4589	0.7457	0.2513	0.4107		0.2463	0.2935	0.1930	0.0751
	p < 0.001	p < 0.001	p = 0.00	p = 0.002	p < 0.001		p = 0.002	p < 0.001	p = 0.017	p = 0.356
**Landslide**	0.6744	0.3067	0.2872	0.8192	0.3864	0.2463		0.3323	0.1694	−0.2871
	p < 0.001	p < 0.001	p < 0.001	p < 0.001	p < 0.001	p = 0.002		p < 0.001	p = 0.036	p < 0.001
**Epidemic**	0.4347	0.2715	0.2684	0.4043	0.1531	0.2935	0.3323		0.0337	−0.1747
	p < 0.001	p = 0.001	p = 0.001	p < 0.001	p = 0.059	p < 0.001	p < 0.001		p = 0.679	p = 0.031
**Neutral**	0.1804	0.5070	0.0669	−0.0986	0.3969	0.1930	0.1694	0.0337		0.4802
	p = 0.026	p < 0.001	p = 0.412	p = 0.226	p < 0.001	p = 0.017	p = 0.036	p = 0.679		p < 0.001
**Positive**	−0.2394	0.3069	0.0140	−0.5306	0.0583	0.0751	−0.2871	−0.1747	0.4802	
	p = 0.003	p < 0.001	p = 0.864	p < 0.001	p = 0.474	p = 0.356	p < 0.001	p = 0.031	p < 0.001	

Each of the stimuli was numbered from 1 to 200 and the entire set was then divided into two subsets according to the code attributed to each stimulus. Stimuli with the Even codes, containing 10 stimuli for each of the 10 categories of natural disasters, were presented to a group of participants; stimuli with the Odd codes, with the remaining 10 stimuli for each category, were presented to a different group of participants.

Participants received an invitation to take part in an online survey, created and distributed by means of Qualtrics XM (https://www.qualtrics.com/). The test started with the mandatory request to subscribe the informed consent, and then the instructions were presented: participants were informed that 100 images of natural disasters and control stimuli had to be rated according to their valence or arousal level, on a 9-point scale. They were also informed that the task was anonymous, so that no name and surname would be requested, but that some demographic information could be required (although not mandatorily). It was specified that the link could be accessed from any type of device connected to the web (computer, smartphone, tablet), but that it would be preferred to carry it out on a computer for an optimal visualization of the images.

The online workflow was the following: after clicking on the invitation link, the participant was presented with the informed consent. After giving the consent to take part in the study as a volunteer, demographical information were collected, and then the participant was presented with the specific instructions of the task. The subgroups who were asked to express a valence/arousal rating were presented with the valence/arousal response scale, respectively, in order to familiarize with the response keys. In particular, they had to select one of the levels of the Self-Assessment Manikin scale, which pictorially represents the 9 possible levels of the participant’s response (see Data Records paragraph for more details). Stimuli were presented in random order and on the bottom of each stimulus the 9-point response scale was presented. After the rating of each of the 100 stimuli, participants were asked to report their previous personal experience with natural disasters, by choosing for each category the answer that best described their experience. At the end of this section, participants were invited to add their possible comments, otherwise as soon as they left the webpage their responses were directly saved in the online server by Qualtrics XM.

Participants were required to judge each stimulus according to either its valence or its arousal. The valence of a stimulus concerns its hedonic tone along a continuum from very negative to very positive. This means that negative emotions are more associated to negative valence, such as anger, fear and sadness, whereas positive emotions are more associated to positive valence, such as happiness and surprise. Arousal is independent from valence, and it refers to the spontaneous activation elicited by a stimulus. In this context, one stimulus can be associated to a low arousal level, namely a sense of relaxation which is independent from the positive/negative emotional valence, and another one can be associated to a high arousal level because, independently from its valence, it activates the observer. For example, a bleeding wound and a smiling child can transmit a high level or arousal, even if they have an extremely negative and positive valence, respectively. Arousal and valence have been shown to be independent from one another^6,7^, and to be based on the activity of different regions of the prefrontal cortex^8^. These evidence support the circumplex valence-arousal model of emotional stimuli, specifically proposing that the emotional value of a stimulus can be defined as the combination of its valence with its arousal^9,10^. Starting from this model, a large number of studies investigated these two dimensions of emotional stimuli, so much so that they can be considered as the most important and widely shared features by which emotional stimuli are categorized.

**Fig. 1:**
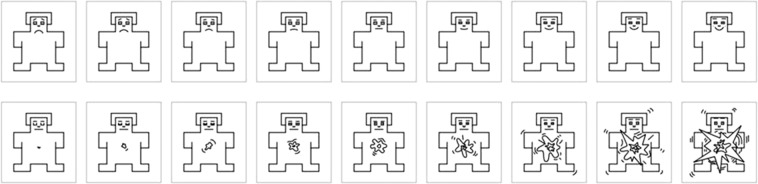
Self-Assessment Manikin. The upper line shows the 9-point valence scale, the lower line shows the 9-point arousal scale.

Responses were collected by using the Self-Assessment Manikin (SAM)^11^, a non-verbal and pictorial assessment technique consisting of a graphic figure, a human-like manikin, depicting a 9-point scale of valence (with a frowning/smiling mouth representing negative/positive valence), or arousal (smaller/larger pointed speech bubble representing lower/higher arousal level). Valence and arousal 9-point scales are shown in Fig. 1. This method has been largely used in studies in which emotional stimuli had to be judged^4,7,8^.

Before starting the task, the participant was presented with the valence or arousal SAM scale (the same as in Fig. 1). The valence group was instructed to select the image that best described the subjective emotional valence elicited by each stimulus, defined as the personal emotional state, by clicking on one of the nine images in the scale. They were specified that the scale comprised 9 points, from the most negative on the left, to the most positive on the right, as represented by the emotional expression of the manikin face. The arousal group was instructed to select the image that best described how much each stimulus was activating, by clicking on one of the nine images in the scale. They were specified that the scale comprised 9 points, from the most relaxed on the left, to the most excited on the right, as represented by the size of the heart of the manikin. The SAM scale was presented below each stimulus, without either numbers or words. Participants were instructed to take all the time they needed to give each response, they were allowed to change a response if they preferred to select a different point of the scale, but they were also instructed that only one response was allowed for each stimulus.

## Data Records

Once data of all participants were collected, the emotional responses were transformed in numerical values, from 1 (most negative/lower arousal rating) to 9 (most positive/higher arousal rating). All raw data are reported online in .csv format and available at FigShare^5^. Mean valence rating, mean arousal rating and reliability index (Cronbach’s alpha values) for each category of stimuli are shown in Table 1, together with the correlation score between valence and arousal ratings for each category.

## Technical Validation

All statistical analyses were carried out by using Statistica 8.0 software (StatSoft, Inc., Tulsa, OK). As reported in Table 1, results showed high levels of internal consistency for each category of stimuli, for both the valence and the arousal scores, with a mean Cronbach’s alpha 0.84 for valence and 0.87 for arousal. Moreover, correlations between valence and arousal scores for each category of stimuli confirmed that valence and arousal are not related to each other, with the sole significant correlation found for the Positive category (when corrected for multiple comparisons the result is not significant).

Furthermore, the ratings recorded for each category were correlated to one another for both valence and arousal, separately: as shown in Table 2, valence scores for the different categories correlated to each other, except for the comparisons Drought-Earthquake, Hailstorm-Epidemic, Neutral-Volcanic activity, Neutral-Earthquake, Neutral-Epidemic, Positive-Volcanic activity, Positive-Hailstorm and Positive-Lightning. Similarly, as shown in Table 3, arousal scores for the different categories significantly correlated to each other, except for the Positive category compared with Fire, Earthquake, Hailstorm and Landslide.

**Table 3:** Arousal scores correlations among the 10 categories of stimuli (*r* and *p* values): all comparisons are significant excepting for: Positive Vs Fire, Earthquake, Hailstorm and Landslide.

*Arousal*	Fire	Drought	Volcanic activity	Earthquake	Hailstorm	Lightning	Landslide	Epidemic	Neutral	Positive
**Fire**		0.6255	0.7505	0.7697	0.7371	0.6594	0.8769	0.6391	0.4447	0.0569
		p < 0.001	p < 0.001	p < 0.001	p < 0.001	p < 0.001	p < 0.001	p < 0.001	p < 0.001	p = 0.399
**Drought**	0.6255		0.5051	0.4624	0.6213	0.4819	0.5556	0.4873	0.7152	0.3649
	p < 0.001		p < 0.001	p < 0.001	p < 0.001	p < 0.001	p < 0.001	p < 0.001	p < 0.001	p < 0.001
**Volcanic activity**	0.7505	0.5051		0.5595	0.5152	0.7883	0.6921	0.4916	0.3287	0.2045
	p < 0.001	p < 0.001		p < 0.001	p < 0.001	p < 0.001	p < 0.001	p < 0.001	p < 0.001	p = 0.002
**Earthquake**	0.7697	0.4624	0.5595		0.6215	0.5539	0.8746	0.5845	0.2827	−0.1073
	p < 0.001	p < 0.001	p < 0.001		p < 0.001	p < 0.001	p < 0.001	p < 0.001	p < 0.001	p = 0.111
**Hailstorm**	0.7371	0.6213	0.5152	0.6215		0.4871	0.7239	0.5110	0.5378	0.1000
	p < 0.001	p < 0.001	p < 0.001	p < 0.001		p < 0.001	p < 0.001	p < 0.001	p < 0.001	p = 0.137
**Lightning**	0.6594	0.4819	0.7883	0.5539	0.4871		0.6721	0.4434	0.3645	0.1778
	p < 0.001	p < 0.001	p < 0.001	p < 0.001	p < 0.001		p < 0.001	p < 0.001	p < 0.001	p = 0.008
**Landslide**	0.8769	0.5556	0.6921	0.8746	0.7239	0.6721		0.6491	0.4281	−0.0312
	p < 0.001	p < 0.001	p < 0.001	p < 0.001	p < 0.001	p < 0.001		p < 0.001	p < 0.001	p = 0.644
**Epidemic**	0.6391	0.4873	0.4916	0.5845	0.5110	0.4434	0.6491		0.4018	0.1875
	p < 0.001	p < 0.001	p < 0.001	p < 0.001	p < 0.001	p < 0.001	p < 0.001		p < 0.001	p = 0.005
**Neutral**	0.4447	0.7152	0.3287	0.2827	0.5378	0.3645	0.4281	0.4018		0.4624
	p < 0.001	p < 0.001	p < 0.001	p < 0.001	p < 0.001	p < 0.001	p < 0.001	p < 0.001		p < 0.001
**Positive**	0.0569	0.3649	0.2045	−0.1073	0.1000	0.1778	−0.0312	0.1875	0.4624	
	p = 0.399	p < 0.001	p = 0.002	p = 0.111	p = 0.137	p = 0.008	p = 0.644	p = 0.005	p < 0.001	

Finally, two separate Analyses of Variance (ANOVA) were carried out to investigate the difference in the judgments expressed by participants for the 10 categories of stimuli. In the first ANOVA, valence ratings for each of the 10 categories were used as the dependent variable, and in the second ANOVA arousal ratings were used as the dependent variable. Categories constituted the within-subject factor in each analysis and post-hoc comparisons were carried out by using Duncan tests (with a significant threshold of *p* < 0.05).

**Fig. 2:**
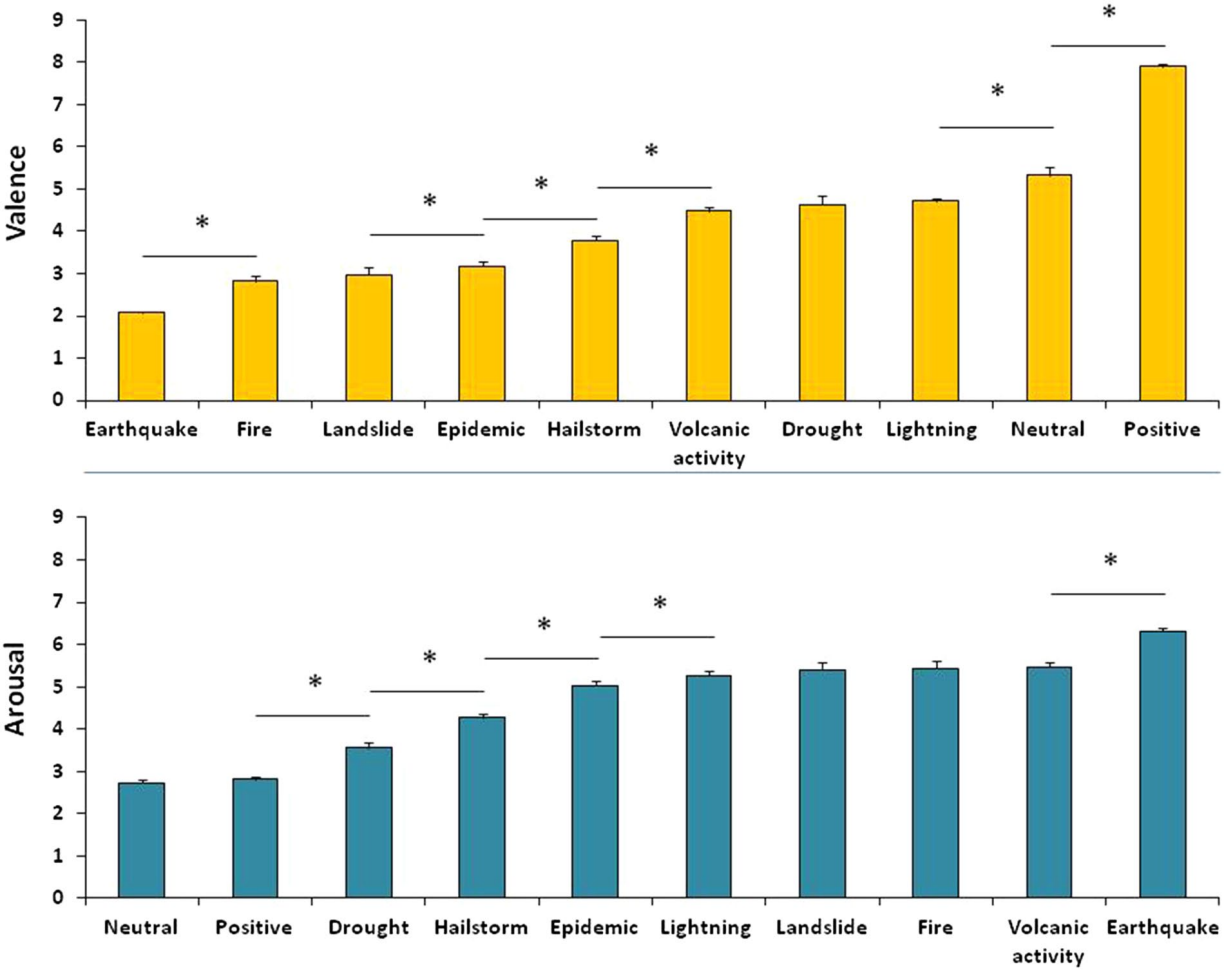
Emotional rating. Upper portion: valence ratings on the 9-point SAM scale for each category of images. Lower portions: arousal ratings on the 9-point SAM scale for each category of images. Bars show standard errors and asterisks show significant comparisons.

As regards valence (*F*_(9, 2421)_ = 515.69, *p* < 0.001, *η*_*p*_^*2*^ = 0.66), post-hoc tests, fully shown in Fig. 2, confirmed that the positive category received the highest evaluations (*p* < 0.001 for all comparisons), followed by the neutral category which differed from all of the other categories (*p* < 0.001 for all comparisons). The lowest judgments were collected for the earthquake category, which was judged as the most negative in valence (*p* < 0.001 for all comparisons).

The ANOVA on Arousal (*F*_(9, 3006)_ = 271.26, *p* < 0.001, *η*_*p*_^*2*^ = 0.45) showed that the earthquake category received the highest arousal evaluations (*p* < 0.001 for all comparisons) and that neutral and positive stimuli received the lowest arousal ratings, without difference between the two (for all of the other comparisons: *p* < 0.001). Figure 2 shows the mean valence and arousal ratings for each category, and asterisks represent the significant comparisons in each ANOVA.

Results showed that different categories of natural disasters correspond to different emotional judgments in terms of both valence and arousal, which are two independent emotional measures, thus confirming the validity of the SAM pictorial scale. As expected, the control categories (neutral and positive stimuli) were judged as more positive and less arousing than all of the other experimental categories, and this pattern of results confirmed the validity of the online test. Importantly, the high reliability levels, as revealed by the Cronbach’s alpha values, further confirmed the internal consistency of the item used.

## Usage Notes

The EaRTH database is a freely available tool for basic and applied research. It can be exploited to investigate both the emotional reaction to natural hazards in the overall and health population, as well as to quantify the hyper-responsiveness to one or more aversive experiences. It could be used to explore differences in emotional reactions among individuals of different geographical provenance, as well as in clinical conditions such as Post-Traumatic Stress Disorder, depression, anxiety and other emotional alterations. Hypothesizing, for instance, the existence of a category-specific traumatic after-effect after being exposed to a specific catastrophic trauma, the demographic information described in figshare Table 1 can constitute a first guide to investigate possible differences among people who experienced that type of event and those who did not. Moreover, also the specific effects of such a personal experience can be associated with the emotional judgements expressed by observers (e.g., having lost a loved one in an earthquake can impact more than having experienced the same earthquake but without serious effects). In this regard, it must be highlighted that some demographic features appear unbalanced among subsamples (a limit of the study, due to the impossibility to balance all the demographic information among subgroups by using the online recruitment), so that data reported in figshare Table 1 are of particular importance in this frame.

Furthermore, the EaRTH database fills a gap in the toolkit of experimental psychology, in which no freely available database on natural disasters exists, despite environmental hazards are among the major current and future challenges to humankind. By means of a large set of naturalistic stimuli (i.e., photographs) and a large sample size, describing also demographic details, EaRTH is a versatile tool for both basic and applied research.

## Data Availability

Codes for the demographic details of the sample, database with stimuli details and sources, and raw data outputs are freely available on FigShare at https://figshare.com/articles/figure/EaRTH_-_The_Environmental_Risks_To_Humans_database/14662173^5^. No custom code was used to generate, process or analyse the data presented in the manuscript.
